# Health-seeking behaviours of young women with sexually transmitted infections: Analysis of the 2014 Ghana Demographic and Health Survey

**DOI:** 10.1371/journal.pone.0277205

**Published:** 2022-11-07

**Authors:** Aaron Asibi Abuosi, Solomon Kwesi Ackon, Emmanuel Anongeba Anaba

**Affiliations:** 1 Department of Public Administration and Health Services Management, University of Ghana Business School, Accra, Ghana; 2 Ankaful Leprosy/ General Hospital, Ankaful, Central Region-Ghana; 3 Department of Population, Family and Reproductive Health, School of Public Health, University of Ghana, Accra, Ghana; University of Botswana, BOTSWANA

## Abstract

**Background:**

Young people are at a disproportionately higher risk for sexually transmitted infections (STIs) due to biological factors, low awareness and limited access to sexual and reproductive health information and services. Untreated STIs can lead to major complications, including HIV, congenital infections, infertility, permanent disability and mortality. This study aimed to identify the salient factors associated with health-seeking behaviours of young women with a history of STIs in Ghana.

**Methods:**

We analysed data from the 2014 Ghana Demographic and Health Survey. In all, we analysed data from a weighted sample of 742 young women with a history of STIs. At the univariate level, frequencies and percentages were computed, while Chi-square analysis was computed at the bivariate level. Both crude and adjusted odds ratios were computed at the multivariable level using binary logistic regression.

**Results:**

The findings showed that the majority (72%) of the participants sought treatment for STIs. Among the participants who sought treatment for STIs (n = 532), 26% sought treatment at a public hospital/polyclinic, 34% sought treatment at a chemical/drug store and 10% self-medicated. Seeking treatment for STIs was significantly associated with older age (20-24yrs), and higher socioeconomic and educational status.

**Conclusion:**

This study demonstrated that majority of the young women sought treatment for STIs. Seeking treatment for STIs was influenced by socio-demographic factors. These findings have implications for sexual and reproductive health policies and interventions in Ghana.

## Background

Sexually transmitted infections (STIs) are infections of the genital tract. STIs are estimated to constitute one of the ten major health challenges in developing countries contributing to the loss of healthy life years [1]. There are over thirty infections transmitted through sexual intercourse. The four most common STIs excluding HIV/AIDS are gonorrhoea, syphilis, chlamydia and trichomoniasis [1]. Ending STIs is a global public health priority. For instance, the revised World Health Organization’s Global Health Sector Strategy seeks to end STIs by 2030 [2]. Notwithstanding, the incidence of STIs remains high across the globe. Over one million STIs are acquired daily and about 498 million new curable STIs occur every year [3]. In 2017, sub-Sahara Africa was the most affected region in the world [4].

Sexual risk behaviours predispose people to STIs [1]. The majority of young people (persons aged 10–24 years) worldwide initiate sexual activities by the age of 20 years [5]. Young people are at a disproportionately higher risk for STIs due to biological factors, and their low levels of awareness as well as limited access to sexual and reproductive health information and services[6]. Young women are at a higher risk of contracting STIs, including HIV [7, 8]. Statistics show that about two million adolescents in the world are living with HIV and over 41.0% of new HIV infections occur among adolescents every year [9]. STIs can either be asymptomatic or symptomatic, yet all STIs can lead to major complications, such as acute morbidities, infertility, permanent disability and mortality if left untreated [10]. In the quest for treatment, persons with STIs may explore different treatment options. Health-seeking behaviour is the process of finding appropriate solutions in response to an illness or health problem [11, 12]. It involves a cycle of activities that individuals go through to correct a perceived health-related issue [13]. Some factors have been identified as either enabling or disrupting the process of seeking healthcare. These include patient characteristics such as gender, age, educational level, symptoms of the diseases, and health care services-related factors such as accessibility, cost and quality of care [13, 14]. Moreover, sociocultural factors such as gender roles and norms and stigma predict health-seeking behaviours [15].

In Ghana, like most developing countries, persons with STIs seek treatment or advice through various avenues, including public or private health facilities, traditional/herbal practitioners, faith healers and self-medication [4]. An important tool in effective STI control is to understand the health-seeking behaviours of people with STIs and the factors that influence their behaviours [12]. Although Ghana has a youthful population, with 55.4% of the population being below 25 years [16], little is known about the health-seeking behaviours of young women with a history of STIs. Prior studies focused on males or women in Accra. Also, existing studies adopted qualitative designs, while the quantitative studies were not peer-reviewed [4, 11, 17, 18]. To the best of the authors’ knowledge, this is the maiden study in Ghana to explore young women’s health-seek behaviours regarding STIs using nationally representative data. The findings of this study would be useful in designing reproductive health communication and advocacy interventions as well as influencing policy initiatives for promoting health-seeking behaviours among young people. This study aimed to identify salient factors influencing the health-seeking behaviours of Ghanaian young women with a history of STIs, using data from the 2014 Ghana Demographic and Health Survey (GDHS).

## Methods

### Data source

Secondary data was obtained from the Demographic and Health Survey program through a formal request via https://dhsprogram.com/. Permission to the anonymized data was granted. The data was downloaded and stored on a password-protected personal computer. We analysed data from the individual recode file’ of the 2014 Ghana Demographic and Health Survey. The 2014 GDHS collected data from women of reproductive age (15–49 years) across all the regions of Ghana. The enumerators collected data regarding demographics, population and health indicators, including a history of STIs. The sample size determination for the Demographic and Health Survey was based on the following estimates: design effect, estimated proportion, desired relative standard error, individual response rate, household gross response rate and the number of eligible individuals per household. The selection of participants was done using a two-stage sampling procedure. The initial stage of the selection comprised selecting 427 enumeration areas, while the second stage comprised selecting 30 households from every enumeration area proportional to size. The sampling frame was the 2010 Population and Housing Census list. Details about the sampling are provided elsewhere [19]. Data were collected by trained enumerators using a paper questionnaire. The enumerators visited the residence of selected participants and administered the questionnaire after seeking their informed consent. Officials from Ghana Statistical Service and Ghana Health Service supervised the data collection process. Data collection lasted from early September to mid-December 2014. Data collection was done across the formal ten administrative regions of Ghana. The enumerators successfully interviewed 9,396 women between the ages of 15 and 49 years. The data were entered into password-protected computers by trained field editors and later transferred to a central office through the secured internet file streaming system.

In this study, we focused on young women (15–24 years), hence 6,069 women who were outside the age group of 15–24 years were dropped from the dataset. Also, we were interested in young women who had a history of STIs, hence a total of 4,047 young women who had no STIs history were dropped from the dataset. In all, we analysed data from a weighted sample of 742 young women with a history of STIs. The 2014 GDHS obtained written informed consent from the caregivers of minors (15–17 years) and adults. The Ghana Health Service Ethics Review Committee approved the protocol of the 2014 GDHS. Further information about the 2014 GDHS is provided in the full report [19].

### Definition of variables

#### Outcome variable

The outcome variable in this study was seeking treatment for STIs (*The last time you had STIs*, *did you seek any kind of advice or treatment*?), which was coded as (0 = No and 1 = Yes). The participants sought treatment at public and private health facilities as well as other outlets. Public health facilities included government hospital/polyclinic = 1, government health centres/clinic = 2, and health post/community health planning and service = 3. The private health facilities included private hospital/clinic = 1, private stand-alone testing center = 2, pharmacy = 3, drug store = 4, maternity home = 5 and other = 6. The other outlets of treatment included home = 1 and other sources = 2.

#### Independent variables

The independent variables in this study comprised socio-demographic factors, exposure to the mass media and barriers to accessing healthcare. The socio-demographic variables included age, educational status, religion, wealth index and type of place of residence. Exposure to the mass media was measured using three items, including frequency of watching television, listening to a radio and reading newspaper/magazine, all coded as (0 = not at all, 1 = least than once a week, and 2 = at least once a week). Barriers to accessing healthcare were measured using four items (*When you are sick and want to get medical advice or treatment*, *is each of the following a big problem or not*? *1) Getting permission to go to the doctor*? *2) Getting money needed for advice or treatment*? *3) The distance to the health facility*? *4) Not wanting to go alone*?*)*. All four items under this dimension were dichotomous variables coded as (1 = big problem and 2 = not a big problem). Health insurance coverage was also measured using a single item (*Are you covered by any health insurance*?), coded as (1 = yes and 2 = no).

### Statistical analysis

Data were analysed with the aid of Stata/SE, version 16 (StataCorp, College Station, Texas, USA). The analytical data set included data from participants aged 15–24 years with a history of STIs. Age of respondent and religion were re-coded and ‘don’t know’ responses were dropped. We adjusted for the sampling weights, clustering, and stratification (complex survey design) using the ‘*svy*’ command, which fits statistical models for complex survey data by adjusting the results of a command for survey settings identified through ‘*svyse*t’. The data were analysed in three stages, including univariate, bivariate and multivariable levels. At the univariate level, frequency and percentage were computed, while Chi-square analysis was computed at the bivariate level. Both crude and adjusted odds ratios were computed at the multivariable level using binary logistic regression. Statistical significance was reported at the 0.05 significance level.

## Results

### Participant characteristics

The results showed that more than half (68%) of the participants were between the ages of 20–24 years. The majority (70%) of the participants had attained senior high school education. In terms of socio-economic status, the majority (60%) of the participants were in the middle class or below. Moreover, 83% of the participants professed Christianity and 56% lived in urban areas. Less than half of the participants (45%) were not covered by health insurance and only 29% of them complained that distance to the health facility was a big problem. Exactly half (50%) of the participants had a big problem with getting money for treatment, whereas the majority (92%) of the participants did not have difficulty getting permission for treatment. Similarly, the majority (84%) of the participants did indicate that going alone for treatment was not a big problem. Also, 75% of participants did not read newspapers, 16% did not listen to the radio and 22% did not watch television (Table 1).

**Table 1:** Participant characteristics (n = 742).

Characteristic	Frequency	Percentage
**Age (years)**		
15–19	240	32
20–24	502	68
**Education status**		
No education	64	9
Primary	126	17
Senior high	517	70
Higher	35	4
**Wealth index**		
Poorest	130	17
Poorer	116	16
Middle	198	27
Richer	174	23
Richest	124	17
**Areas of residence**		
Urban	414	56
Rural	328	44
**Religion**		
Christianity	614	83
Other religion	128	17
**Covered by health insurance**		
No	334	45
Yes	408	55
**Distance to a health facility**		
Big problem	216	29
Not a big problem	526	71
**Getting permission to go for treatment**		
Big problem	59	8
Not a big problem	683	92
**Getting money for treatment,**		
Big problem	369	50
Not a big problem	372	50
**Not wanting to go alone.**		
Big problem	120	16
Not a big problem	622	84
**Frequency of reading newspaper**		
Not at all	560	75
Less than once a week	116	16
At least once a week	65	9
**Frequency of listening to the radio**		
Not at all	117	16
Less than once a week	249	33
At least once a week	376	51
**Frequency of watching television**		
Not at all	159	22
Less than once a week	210	28
At least once a week	373	50

### Health-seeking behaviours of young women with STIs

The findings showed that the majority (n = 532; 72%) of the participants sought treatment for STIs. Among the participants who sought treatment for STIs (n = 532), 34% sought treatment at a chemical shop/drug store, 26% sought treatment at a government hospital/polyclinic and 10% self-medicated (Table 2).

**Table 2:** Health-seeking behaviours of young women with STIs (n = 742).

Items	Frequency	Percentage
**Sought treatment for** STIs		
No	210	28
Yes	532	72
**Government hospital/polyclinic**		
No	396	74
Yes	136	26
**Government health centre/clinic**		
No	472	89
Yes	60	11
**Health post/community health planning and services**		
No	521	98
Yes	11	2
**Private hospital/clinic/doctor**		
No	493	93
Yes	39	7
**Private stand-alone voluntary counselling and testing centre**		
No	527	99
Yes	5	1
**Pharmacy**		
No	489	92
Yes	43	8
**Chemical/drug store**		
No	352	66
Yes	180	34
**Maternity home**		
No	528	99
Yes	4	1
**Other private**		
No	530	99
Yes	2	1
**Self-medicate**		
No	481	90
Yes	51	10
**Other sources**		
No	507	95
Yes	25	5

### Association between participant characteristics and seeking treatment for STIs

The study found that age, wealth index, educational status and area of residence were significantly associated with seeking treatment for STIs among young women (p < 0.05). The study also found a significant association between the frequency of listening to the radio, reading newspapers, watching television and getting permission for treatment, and seeking treatment for STIs (p < 0.05). Moreover, 77% of young women in urban areas sought treatment compared to 65% of those in rural areas. Four in ten young women aged 15–19 years, with no formal education or in the poorest wealth index did not seek treatment for STIs. Also, four in ten young women who had problems seeking permission for treatment, who did not listen to the radio or did not watch television failed to seek treatment for STIs (Table 3).

**Table 3:** Association between participant characteristics and seeking treatment for STIs (n = 742).

Characteristics	*Did you seek treatment for STIs*?		
	No n (%)	Yes n (%)	Chi-square
**Age (years)**			
15–19	102(42)	138(58)	30.68 **
20–24	108(22)	395(78)	
**Education status**			
No education	29(44)	36(56)	
Primary	55(43)	72(57)	8.57 **
Senior high	122(24)	395(76)	
Higher	5(13)	30(87)	
**Wealth index**			
Poorest	56(43)	74(57)	
Poorer	39(34)	77(66)	
Middle	60(30)	138(70)	5.48 **
Richer	27(16)	147(84)	
Richest	27(22)	97(78)	
**Areas of residence**			
Urban	95(23)	319(77)	9.03 **
Rural	115(35)	214(65)	
**Religion**			
Christianity	165(27)	449(73)	2.51
Other religion	45(35)	84(65)	
**Covered by health insurance**			
No	108(32)	226(68)	3.39
Yes	102(25)	306(75)	
**Distance to a health facility**			
Big problem	68(32)	148(68)	1.11
Not a big problem	142(27)	384(73)	
**Getting permission to go for treatment**			
Big problem	25(42)	34(58)	3.97
Not a big problem	185(27)	498(73)	
**Getting money for treatment,**			
Big problem	116(31)	253(69)	1.95
Not a big problem	94(25)	280(75)	
**Not wanting to go alone.**			
Big problem	35(29)	85(71)	0.02
Not a big problem	175(28)	447(72)	
**Frequency of reading newspaper**			
Not at all	170(30)	390(70)	
Less than once a week	20(18)	95(82)	3.01
At least once a week	18(28)	47(72)	
**Frequency of listening to the radio**			
Not at all	53(46)	63(54)	
Less than once a week	72(29)	177(71)	7.92 **
At least once a week	84(22)	293(78)	
**Frequency of watching television**			
Not at all	65(41)	95(59)	
Less than once a week	64(31)	146(69)	7.35 **
At least once a week	81(22)	292(78)	

* p-value < 0.05

** p-value < 0.01

### Binary logistic regression analysis

In the crude analysis, it was found that age, educational status, wealth index, area of residence, getting permission to go for treatment, frequency of reading newspapers, frequency of listening to the radio and the frequency of watching television were significant predictors of seeking treatment for STIs among young women (p ≤ 0.05). For instance, young women between the ages of 20–24 were about three times more likely (COR = 2.68; 95% CI = 1.88–3.84) to seek treatment for STIs compared to those aged 15–19 years. Also, young women who had higher education had higher odds (COR = 5.19; 95% CI = 1.55–17.19) of seeking treatment for STIs compared to young women with no formal education. Young women who had no difficulty in getting permission to go for treatment were two times more likely (COR = 1.94; 95% CI = 0.99–3.78) to seek treatment compared to those with difficulty in getting permission to go for treatment (Table 4).

**Table 4:** Determinants of seeking treatment for STIs among young women in Ghana (n = 742).

Covariates	COR 95% CI	AOR 95% CI
**Age (years)**		
15–19	1(ref)	1(ref)
20–24	2.68(1.88–3.84) **	2.79(1.82–4.29) **
**Education status**		
No education	1(ref)	1(ref)
Primary	1.04(0.55–1.99)	1.17(0.60–2.25)
Senior high	2.58(1.40–4.76) **	2.22(1.16–4.25) *
Higher	5.17(1.55–17.19) **	2.83(0.74–10.78)
**Wealth index**		
Poorest	1(ref)	1(ref)
Poorer	1.48(0.83–2.66)	1.96(0.64–2.23)
Middle	1.73(0.96–3.10)	1.14(0.57–2.26)
Richer	4.13(2.21–7.75) **	2.15(1.01–4.56) *
Richest	2.70(1.28–5.68) **	1.29(0.45–3.64)
**Areas of residence**		
Urban	1(ref)	1(ref)
Rural	0.55(0.38–0.81) **	0.86(0.51–1.47)
**Religion**		
Christianity	1(ref)	1(ref)
Other religion	0.68(0.42–1.09)	0.97(0.59–1.60)
**Covered by health insurance**		
No	1(ref)	1(ref)
Yes	1.44(0.97–2.13)	1.46(0.96–2.22)
**Distance to a health facility**		
Big problem	1(ref)	1(ref)
Not a big problem	1.24(0.82–1.88)	1.08(0.65–1.80)
**Getting permission to go for treatment**		
Big problem	1(ref)	1(ref)
Not a big problem	1.94(0.99–3.78)	1.90(0.97–3.71)
**Getting money for treatment,**		
Big problem	1(ref)	1(ref)
Not a big problem	1.36(0.88–2.09)	0.99(0.59–1.64)
**Not wanting to go alone.**		
Big problem	1(ref)	1(ref)
Not a big problem	1.04(0.58–1.86)	0.67(0.35–1.26)
**Frequency of reading newspaper**		
Not at all	1(ref)	1(ref)
Less than once a week	2.05(1.17–3.60) *	1.31(0.74–2.33)
At least once a week	1.13(0.56–2.28)	0.58(0.27–1.26)
**Frequency of listening to the radio**		
Not at all	1(ref)	1(ref)
Less than once a week	2.06(1.19–3.56) *	1.70(0.98–2.97)
At least once a week	2.94(1.72–5.02) **	1.79(1.00–3.22) *
**Frequency of watching television**		
Not at all	1(ref)	1(ref)
Less than once a week	1.54(0.93–2.56)	1.12(0.60–2.07)
At least once a week	2.45(1.53–3.93) **	0.68(0.61–2.05)

* p-value < 0.05

** p-value < 0.01

After simultaneously adjusting for the covariates, age, educational status, wealth index and frequency of listening to radio emerged as significant predictors of seeking treatment for STIs among young women (p ≤ 0.05). For instance, young women between the ages of 20–24 had higher odds (AOR = 2.79; 95% CI = 1.82–4.29) of seeking treatment compared to young women aged 15–19 years. In addition, young women who had senior high school education were two times more likely (AOR = 2.22; 95% CI = 1.16–4.25) to seek treatment compared to young women who had no formal education. Also, young women in the richer wealth quintile had a higher likelihood (AOR = 2.15; 95% CI = 1.01–4.56) of seeking treatment for STIs compared to those in the poorest wealth quintile. Finally, young women who listened to the radio at least once a week had higher odds (AOR = 1.79; 95% CI: 1.00–3.22) of seeking treatment for STIs compared with those who did not listen to the radio (Table 4).

## Discussion

The key findings of this study were that majority of the participants sought treatment for STIs. A significant minority of the participants sought treatment at a chemical/drug store or self-medicated. It was found that seeking treatment for STIs was significantly associated with age, educational status and socioeconomic status. However, a significant minority of the participants did not seek treatment for STIs.

We found that seven in ten young women sought treatment for STIs. This finding is supported by recent studies [20, 21]. For instance, a study in Ghana demonstrated that seven in ten persons who had STIs sought treatment [4]. The prevalence in this study is lower than the prevalence in Nkomazi East, South Africa, where 100% of the participants sought treatment for STIs [21]. However, the prevalence in this study is higher than in an earlier study in Ghana where three in ten women with STIs sought treatment [11], and in Tamil Nadu, India, where 51% of married women with STIs sought treatment [22]. This finding suggests that young women of contemporary times are more likely to seek treatment, perhaps due to improved access to health information through the mass media. The differences in the findings may be due to variations in contextual factors and access to health information and treatment centres.

In addition, this study showed that a considerable proportion (34%) of young women sought treatment at a chemical/drug store and 10% self-medicated. Similar studies in Nigeria showed that a substantial proportion of people with STIs sought treatment from patent drug vendors or used over-the-counter drugs [14, 20, 23]. This finding is understandable because chemical/drug stores are easily accessible and available in most Ghanaian communities. Community pharmacies are the first point of contact for most Ghanaians who have drug-related health problems [24]. The prevalence of self-medication in this study is higher than what was found in Iran (8%) [25]. The differences in findings may be attributed to disparities in contextual factors. The fact that a considerable proportion of young women did not seek treatment at health facilities should be a cause for concern for stakeholders. There is evidence to show that individuals who buy over-the-counter drugs to treat STIs mostly receive inappropriate dosages and advice [26]. This can lead to overdose, antimicrobial resistance, further complications and death. Inappropriate use of antibiotics, including overdose and self-medication, increases the spread of antimicrobial resistance [27], which is now a global health threat [28, 29]. There is a need to enforce policies and guidelines to regulate the activities of chemical/drug store operators as well as educate young women about the dangers of self-medication.

This study demonstrated that age was significantly associated with seeking treatment for STIs. Young women aged 20–24 years had higher chances of seeking treatment for STIs compared to those aged 15–19 years. Previous studies have demonstrated that age is a major determinant of health-seeking behaviours among STI clients [30, 31]. This finding may be due to the fact that young adults (20–24 years) do not require the consent of their parents/caregivers before seeking health care or medical advice. In addition, women aged 20–24 years are more likely to access antenatal, delivery and postnatal care, hence they might have sought STIs treatment as well [22].

The findings revealed that young women with secondary education were two times more likely to seek treatment for STIs compared to those with no formal education. Similar studies have corroborated this finding [30–32]. For example, a similar study in North India found that STI clients who were literate were thirty-seven times more likely to seek treatment compared to clients who were illiterate [32]. A plausible reason is that persons who are educated may be more aware and knowledgeable about STIs symptoms and treatment centres [30, 32]. Research has shown that individuals who are knowledgeable about STIs are more likely to find testing services acceptable and not stigmatizing [33]. This implies that promoting girls’ inclusion in formal education can help improve their health and well-being.

Further, socio-economic status emerged as a significant predictor of seeking treatment for STIs. Young women in wealthy households were two times more likely to seek treatment for STIs compared to those in poor households. This finding is expected and has been supported by prior studies [30, 34]. The reason is that young women in wealthy households probably can afford transportation costs to visit health facilities. Further, persons in wealthy households are more likely to afford the cost of healthcare as well as being covered by health insurance schemes. Moreover, a higher socioeconomic status may increase independence and decision-making power, which are crucial in making health decisions [22].

It was also interesting to find that a significant minority (28%) of young women did not seek treatment for STIs. This finding is lower than a study in India (49%)[35] but higher than another study in South Africa [21]. Evidence shows that women may not seek treatment for STIs because of financial constraints, perceived discrimination, severity and shyness of genital examination [36]. These findings imply that younger women aged 15–19 years, those with no formal education and those in poor households may not seek treatment for STIs. These young women may therefore be at greater risk for complications arising from untreated STIs and may also be more likely to infect their partners. Therefore, we suggest that sexual and reproductive health programmes should focus on vulnerable populations, including young women from low socioeconomic backgrounds. It is also recommended that interventions should focus on bridging the socio-economic inequalities. The Ministry of Health and related agencies should intensify public education on STIs and the danger associated with the inappropriate use of antibiotics.

### Strengths and limitations of the study

The findings of this study provide relevant information for sexual and reproductive health policies and programmes. A major strength of this study is that the data were nationally representative, therefore the findings can be generalized to all young women in Ghana. Also, few studies have explored the subject matter in Africa, hence this study makes a modest contribution to the literature on the subject matter. Notwithstanding, this study is not devoid of limitations. The findings of the study must be interpreted with caution due to research biases, including social desirability bias and recall bias. Also, cross-sectional designs cannot establish causality or explain the reasons behind the findings. In addition, we did not distinguish between young women who sought treatment from health facilities and those who self-medicated or sought no treatment. Therefore, future studies should adopt research designs that can address these limitations.

## Conclusion

This study has demonstrated that a significant minority of young women in Ghana did not seek treatment for STIs. Among those who sought treatment, a considerable proportion utilized chemical/drug stores. Seeking treatment for STIs was influenced by age, education and socioeconomic status. These findings have implications for improving the health-seeking behaviours of young women in Ghana. Interventions that seek to improve health-seeking behaviours among young women with STIs should prioritize adolescent girls (15–19 years) and young women of low socio-economic backgrounds. This can help reduce the burden of STI-related complications and deaths among young women in Ghana.
